# 4-[(*E*)-(Hy­droxy­imino)­meth­yl]-*N*,*N*-di­methyl­anilinium chloride

**DOI:** 10.1107/S1600536812020211

**Published:** 2012-05-12

**Authors:** T. Uma Devi, G. Kalpana, S. Priya, K. Ravikumar, S. Selvanayagam

**Affiliations:** a Department of Physics, Government Arts College for Women, Pudukkottaii 622 001, India; b Department of Physics, Shivani Institute of Technology, Tiruchirappalli 620 009, India; c Department of Physics, Cauvery College for Women, Tiruchirappalli 620 018, India; d Laboratory of X-ray Crystallography, Indian Institute of Chemical Technology, Hyderabad 500 007, India; e Department of Physics, Kalasalingam University, Krishnankoil 626 126, India

## Abstract

In the title compound, C_9_H_13_N_2_O^+^·Cl^−^, the cation, apart from the methyl groups, is almost planar, with a maximum deviation of 0.040 (1) Å; the methyl C atoms deviate by 0.389 (2) and −1.247 (1) Å, from the mean plane. In the crystal, cations and anions associate through C—H⋯Cl hydrogen bonds, forming a helical arrangement. In addition, inter­molecular O—H⋯Cl, N—H⋯Cl and C—H⋯N inter­actions are observed.

## Related literature

For general background to hydroxyl­amine derivatives, see: Kataoka *et al.* (2002 ▶); Haldimann *et al.* (2011 ▶) and to benzalde­hyde derivatives, see: Haraguchi *et al.* (2011 ▶); Johnston *et al.* (2011 ▶); Zhang *et al.* (2011 ▶). For a related structure, see: Bachechi & Zambonelli (1972 ▶).
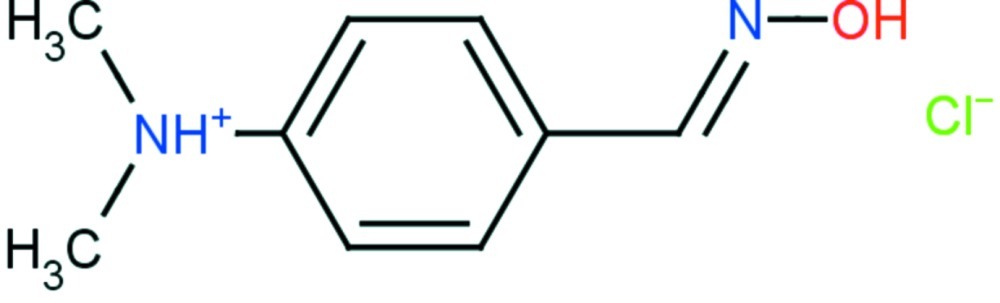


## Experimental

### 

#### Crystal data

C_9_H_13_N_2_O^+^·Cl^−^
*M* *_r_* = 200.66
Monoclinic, 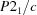

*a* = 11.2696 (10) Å
*b* = 11.7093 (10) Å
*c* = 7.6961 (7) Å
β = 90.108 (2)°
*V* = 1015.57 (16) Å^3^
*Z* = 4
Mo *K*α radiation
μ = 0.34 mm^−1^
*T* = 292 K
0.24 × 0.20 × 0.19 mm

#### Data collection

Bruker SMART APEX CCD area-detector diffractometer
11453 measured reflections
2405 independent reflections
2240 reflections with *I* > 2σigma(*I*)
*R*_int_ = 0.025

#### Refinement

*R*[*F* ^2^ > 2σ(*F* ^2^)] = 0.031
*wR*(*F* ^2^) = 0.093
*S* = 1.07
2405 reflections
125 parameters
1 restraint
H atoms treated by a mixture of independent and constrained refinement
Δρ_max_ = 0.23 e Å^−3^
Δρ_min_ = −0.20 e Å^−3^

### 

Data collection: *SMART* (Bruker, 2001 ▶); cell refinement: *SAINT* (Bruker, 2001 ▶); data reduction: *SAINT*; program(s) used to solve structure: *SHELXS97* (Sheldrick, 2008 ▶); program(s) used to refine structure: *SHELXL97* (Sheldrick, 2008 ▶); molecular graphics: *ORTEP-3* (Farrugia, 1997 ▶) and *PLATON* (Spek, 2009 ▶); software used to prepare material for publication: *SHELXL97* and *PLATON*.

## Supplementary Material

Crystal structure: contains datablock(s) I, global. DOI: 10.1107/S1600536812020211/zq2166sup1.cif

Structure factors: contains datablock(s) I. DOI: 10.1107/S1600536812020211/zq2166Isup2.hkl

Supplementary material file. DOI: 10.1107/S1600536812020211/zq2166Isup3.cml

Additional supplementary materials:  crystallographic information; 3D view; checkCIF report

## Figures and Tables

**Table 1:** Hydrogen-bond geometry (Å, °)

*D*—H⋯*A*	*D*—H	H⋯*A*	*D*⋯*A*	*D*—H⋯*A*
O1—H1⋯Cl1^i^	0.82	2.34	3.147 (1)	167
N1—H1*N*⋯Cl1^ii^	0.91 (1)	2.14 (1)	3.040 (1)	173 (1)
C6—H6⋯N2^iii^	0.93	2.59	3.516 (2)	173
C7—H7⋯Cl1^iv^	0.93	2.81	3.697 (1)	160
C9—H9*B*⋯Cl1^v^	0.96	2.81	3.713 (2)	158

Symmetry codes: (i) 
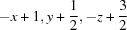
; (ii) 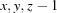; (iii) 
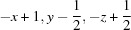
; (iv) 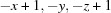; (v) 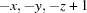.

**Fig. 1.**
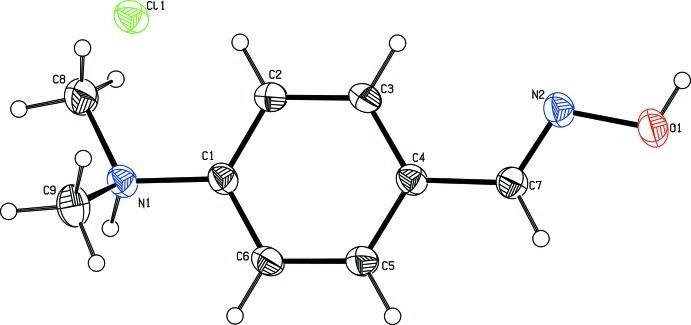
The molecular structure of the title compound. Displacement ellipsoids are drawn at the 30% probability level

**Fig. 2.**
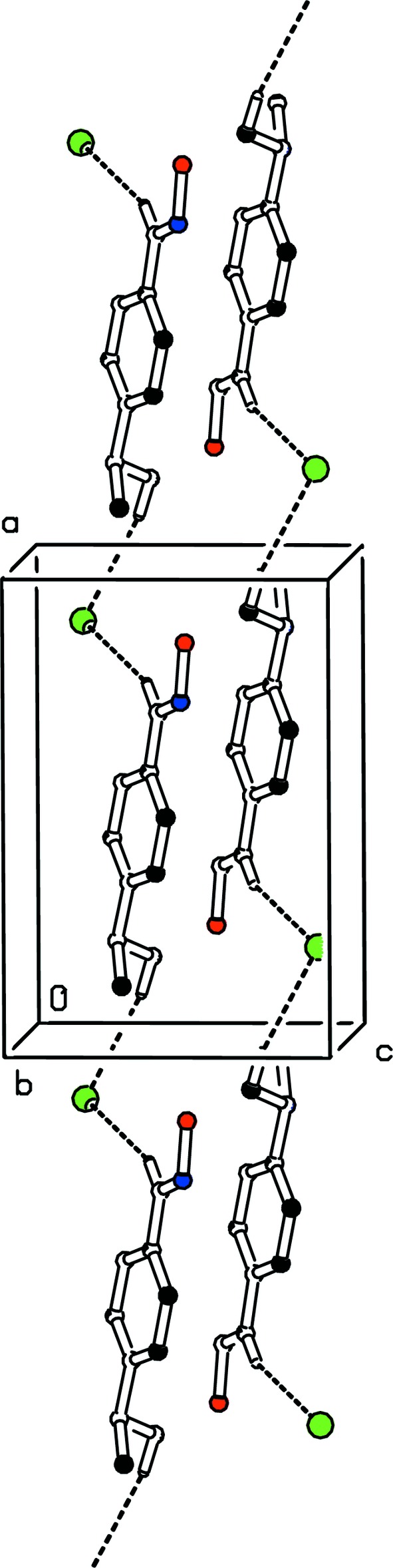
Molecular packing of the title compound, viewed along the b axis (H-bonds are shown as dashed lines). For the sake of clarity, H atoms which are not involved in hydrogen bonds have been omitted.

**Fig. 3.**
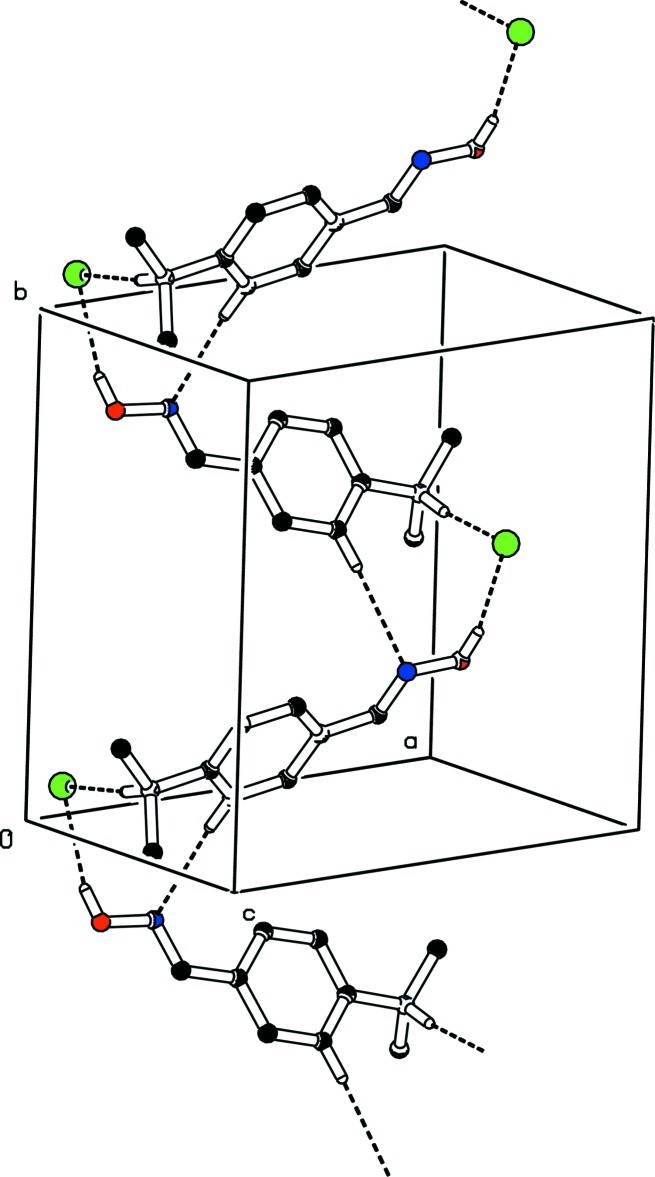
Molecular packing of the title compound, viewed along the c axis (H-bonds are shown as dashed lines). For the sake of clarity, H atoms which are not involved in hydrogen bonds have been omitted.

## supplementary crystallographic information

### Comment

Hydroxylamine derivatives possess anti-inflammatory and anti-allergic activities (Kataoka *et al.*, 2002). The novel hydroxylamine derivative NG-094 suppresses polyglutamine protein toxicity in Caenorhabditis elegans (Haldimann *et al.*, 2011). The benzaldehyde-modified starches and starch components have significantly higher water solubility than their native counterparts (Johnston *et al.*, 2011). Benzaldehyde derivatives possess antibacterial (Zhang *et al.*, 2011) and antitrypanasomal (Haraguchi *et al.*, 2011) activities. In continuation of our work, we have undertaken the crystal structure determination of the present complex, and the results are presented here.

The X-ray study confirmed the molecular structure of the title compound as illustrated in Fig. 1. Atom H1N was located from a difference Fourier map and refined freely. The protonation on the N1 site of the cation is also confirmed from the C1—N1 bond distance of 1.4777 (14) Å in comparison with the C—N bond distance of 1.380 (4) Å observed in the crystal structure of the neutral α-*p*-dimethylaminobenzaldoxime (Bachechi & Zambonelli, 1972). The bond distance N2—C7 of 1.267 (2) Å confirms the double bond character. The cation is almost planar with a maximum deviation of -0.040 (1) Å for atom C3 and the two methyl carbon atoms C8 and C9 deviate by 0.389 (2) and -1.247 (1) Å, respectively, from this plane.

Cations and anions associate through intermolecular C—H···Cl hydrogen bonds. These two hydrogen bonds are run in opposite direction of the *ab* plane forming a helical shape arrangement (Fig. 2 and Table 1). Intermolecular O—H···Cl, N—H···Cl and C—H···N interactions are also observed in the crystal structure (Fig. 3). In addition, the molecules are also connected by C—H···π interactions, the H3 atom (bound to C3) is at 2.87 Å from the centroid Cg1^i^ of the phenyl ring (symmetry code *i* = x, 1/2 - y, 1/2 + z), with a C3—H3···Cg1^i^ angle of 135° and a C3···Cg1 distance of 3.589 (2) Å.

### Experimental

Commercially availbale hydroxylamine hydrochloride with *p*-dimethyl amino benzaldehyde was taken in equimolar ratio, were dissolved in double ethanol and stirred to yield a homogeneous mixture. The solution was allowed to evaporate at room temperature which yielded a brown crystalline salt. Single crystals were grown by slow evaporation from DMF.

### Refinement

Atom H1N was located from a difference Fourier map and refined with a distance restraint of 0.89 (2) Å. The remaining H atoms were positioned geometrically and were treated as riding on their parent C and O atoms, with C—H = 0.93 Å and *U*_iso _= 1.2*U*_eq_(C) for aromatic H atoms, with C—H = 0.96 Å and *U*_iso _= 1.5*U*_eq_(C) for methyl H atoms, and with O—H = 0.82 Å and *U*_iso _= 1.2*U*_eq_(O).

### Crystal data

**Table:**

C_9_H_13_N_2_O^+^·Cl^−^	*F*(000) = 424
*M**_r_* = 200.66	*D*_x_ = 1.312 Mg m^−^^3^
Monoclinic, *P*2_1_/*c*	Mo *K*α radiation, λ = 0.71073 Å
Hall symbol: -P 2ybc	Cell parameters from 7257 reflections
*a* = 11.2696 (10) Å	θ = 2.3–26.6°
*b* = 11.7093 (10) Å	µ = 0.34 mm^−^^1^
*c* = 7.6961 (7) Å	*T* = 292 K
β = 90.108 (2)°	Needle, brown
*V* = 1015.57 (16) Å^3^	0.24 × 0.20 × 0.19 mm
*Z* = 4	

### Data collection

**Table:**

Bruker SMART APEX CCD area-detector diffractometer	2240 reflections with *I* > 2σigma(*I*)
Radiation source: fine-focus sealed tube	*R*_int_ = 0.025
Graphite monochromator	θ_max_ = 28.0°, θ_min_ = 1.8°
ω scans	*h* = −14→14
11453 measured reflections	*k* = −15→15
2405 independent reflections	*l* = −9→10

### Refinement

**Table:**

Refinement on *F*^2^	Primary atom site location: structure-invariant direct methods
Least-squares matrix: full	Secondary atom site location: difference Fourier map
*R*[*F*^2^ > 2σ(*F*^2^)] = 0.031	Hydrogen site location: inferred from neighbouring sites
*wR*(*F*^2^) = 0.093	H atoms treated by a mixture of independent and constrained refinement
*S* = 1.07	*w* = 1/[σ^2^(*F*_o_^2^) + (0.0536*P*)^2^ + 0.1829*P*] where *P* = (*F*_o_^2^ + 2*F*_c_^2^)/3
2405 reflections	(Δ/σ)_max_ < 0.001
125 parameters	Δρ_max_ = 0.23 e Å^−^^3^
1 restraint	Δρ_min_ = −0.20 e Å^−^^3^

### Special details

**Table:**

Geometry. All esds (except the esd in the dihedral angle between two l.s. planes) are estimated using the full covariance matrix. The cell esds are taken into account individually in the estimation of esds in distances, angles and torsion angles; correlations between esds in cell parameters are only used when they are defined by crystal symmetry. An approximate (isotropic) treatment of cell esds is used for estimating esds involving l.s. planes.
Refinement. Refinement of F^2^ against ALL reflections. The weighted R-factor wR and goodness of fit S are based on F^2^, conventional R-factors R are based on F, with F set to zero for negative F^2^. The threshold expression of F^2^ > 2sigma(F^2^) is used only for calculating R-factors(gt) etc. and is not relevant to the choice of reflections for refinement. R-factors based on F^2^ are statistically about twice as large as those based on F, and R- factors based on ALL data will be even larger.

### Fractional atomic coordinates and isotropic or equivalent isotropic displacement parameters (Å^2^)

**Table:**

	*x*	*y*	*z*	*U*_iso_*/*U*_eq_	
Cl1	0.16123 (3)	0.02780 (2)	0.86101 (4)	0.04216 (12)	
O1	0.81494 (8)	0.28038 (8)	0.48301 (14)	0.0490 (2)	
H1	0.8321	0.3407	0.5309	0.073*	
N1	0.17646 (8)	0.07318 (8)	0.24932 (13)	0.0370 (2)	
H1N	0.1744 (14)	0.0531 (13)	0.1354 (18)	0.050 (4)*	
N2	0.69124 (9)	0.27159 (9)	0.46814 (14)	0.0405 (2)	
C1	0.30082 (10)	0.10295 (10)	0.29034 (14)	0.0354 (2)	
C2	0.32816 (11)	0.20083 (11)	0.38181 (18)	0.0472 (3)	
H2	0.2683	0.2494	0.4197	0.057*	
C3	0.44580 (11)	0.22569 (11)	0.41636 (18)	0.0471 (3)	
H3	0.4647	0.2913	0.4787	0.057*	
C4	0.53633 (10)	0.15418 (9)	0.35930 (14)	0.0351 (2)	
C5	0.50650 (11)	0.05628 (10)	0.26681 (16)	0.0395 (3)	
H5	0.5661	0.0076	0.2283	0.047*	
C6	0.38908 (11)	0.03048 (9)	0.23151 (16)	0.0400 (3)	
H6	0.3697	−0.0349	0.1689	0.048*	
C7	0.66167 (10)	0.18062 (10)	0.38936 (15)	0.0370 (2)	
H7	0.7199	0.1305	0.3506	0.044*	
C8	0.08882 (12)	0.16749 (13)	0.2724 (2)	0.0568 (4)	
H8A	0.0145	0.1459	0.2209	0.085*	
H8B	0.1179	0.2354	0.2172	0.085*	
H8C	0.0776	0.1818	0.3941	0.085*	
C9	0.13654 (13)	−0.03099 (11)	0.34631 (19)	0.0494 (3)	
H9A	0.1896	−0.0931	0.3223	0.074*	
H9B	0.0577	−0.0512	0.3100	0.074*	
H9C	0.1367	−0.0154	0.4688	0.074*	

### Atomic displacement parameters (Å^2^)

**Table:**

	*U*^11^	*U*^22^	*U*^33^	*U*^12^	*U*^13^	*U*^23^
Cl1	0.04579 (19)	0.03872 (18)	0.04194 (19)	0.00255 (10)	−0.00722 (12)	−0.00218 (10)
O1	0.0383 (5)	0.0437 (5)	0.0649 (6)	−0.0055 (3)	−0.0106 (4)	−0.0029 (4)
N1	0.0367 (5)	0.0376 (5)	0.0367 (5)	−0.0020 (4)	−0.0068 (4)	−0.0022 (4)
N2	0.0368 (5)	0.0381 (5)	0.0467 (5)	−0.0010 (4)	−0.0075 (4)	0.0001 (4)
C1	0.0351 (5)	0.0366 (5)	0.0344 (5)	−0.0020 (4)	−0.0050 (4)	−0.0021 (4)
C2	0.0380 (6)	0.0472 (7)	0.0564 (7)	0.0046 (5)	−0.0034 (5)	−0.0200 (6)
C3	0.0412 (6)	0.0445 (6)	0.0555 (7)	0.0002 (5)	−0.0067 (5)	−0.0213 (6)
C4	0.0376 (5)	0.0343 (5)	0.0334 (5)	0.0002 (4)	−0.0050 (4)	−0.0008 (4)
C5	0.0393 (6)	0.0339 (5)	0.0453 (6)	0.0036 (4)	−0.0021 (5)	−0.0059 (5)
C6	0.0425 (6)	0.0324 (5)	0.0450 (6)	−0.0012 (4)	−0.0047 (5)	−0.0089 (4)
C7	0.0371 (6)	0.0359 (5)	0.0379 (5)	0.0022 (4)	−0.0046 (4)	−0.0004 (4)
C8	0.0409 (7)	0.0507 (7)	0.0786 (10)	0.0058 (6)	−0.0136 (6)	−0.0133 (7)
C9	0.0463 (7)	0.0514 (8)	0.0506 (7)	−0.0096 (5)	−0.0054 (6)	0.0100 (5)

### Geometric parameters (Å, º)

**Table:**

O1—N2	1.4023 (13)	C4—C5	1.3903 (16)
O1—H1	0.8200	C4—C7	1.4640 (15)
N1—C1	1.4777 (14)	C5—C6	1.3838 (17)
N1—C8	1.4924 (17)	C5—H5	0.9300
N1—C9	1.4995 (16)	C6—H6	0.9300
N1—H1N	0.908 (13)	C7—H7	0.9300
N2—C7	1.2698 (15)	C8—H8A	0.9600
C1—C2	1.3796 (16)	C8—H8B	0.9600
C1—C6	1.3842 (16)	C8—H8C	0.9600
C2—C3	1.3827 (18)	C9—H9A	0.9600
C2—H2	0.9300	C9—H9B	0.9600
C3—C4	1.3916 (17)	C9—H9C	0.9600
C3—H3	0.9300		
			
N2—O1—H1	109.5	C6—C5—H5	119.6
C1—N1—C8	115.32 (9)	C4—C5—H5	119.6
C1—N1—C9	111.77 (9)	C5—C6—C1	119.30 (10)
C8—N1—C9	110.08 (11)	C5—C6—H6	120.4
C1—N1—H1N	106.8 (10)	C1—C6—H6	120.4
C8—N1—H1N	106.9 (10)	N2—C7—C4	120.34 (11)
C9—N1—H1N	105.3 (10)	N2—C7—H7	119.8
C7—N2—O1	111.13 (10)	C4—C7—H7	119.8
C2—C1—C6	121.09 (11)	N1—C8—H8A	109.5
C2—C1—N1	121.05 (10)	N1—C8—H8B	109.5
C6—C1—N1	117.86 (10)	H8A—C8—H8B	109.5
C1—C2—C3	119.09 (11)	N1—C8—H8C	109.5
C1—C2—H2	120.5	H8A—C8—H8C	109.5
C3—C2—H2	120.5	H8B—C8—H8C	109.5
C2—C3—C4	121.06 (11)	N1—C9—H9A	109.5
C2—C3—H3	119.5	N1—C9—H9B	109.5
C4—C3—H3	119.5	H9A—C9—H9B	109.5
C5—C4—C3	118.75 (11)	N1—C9—H9C	109.5
C5—C4—C7	119.18 (10)	H9A—C9—H9C	109.5
C3—C4—C7	122.05 (10)	H9B—C9—H9C	109.5
C6—C5—C4	120.72 (11)		
			
C8—N1—C1—C2	14.77 (17)	C3—C4—C5—C6	0.22 (18)
C9—N1—C1—C2	−111.95 (13)	C7—C4—C5—C6	−178.05 (11)
C8—N1—C1—C6	−164.17 (12)	C4—C5—C6—C1	−0.43 (19)
C9—N1—C1—C6	69.11 (14)	C2—C1—C6—C5	0.67 (19)
C6—C1—C2—C3	−0.7 (2)	N1—C1—C6—C5	179.61 (11)
N1—C1—C2—C3	−179.60 (12)	O1—N2—C7—C4	−179.96 (10)
C1—C2—C3—C4	0.5 (2)	C5—C4—C7—N2	177.52 (11)
C2—C3—C4—C5	−0.2 (2)	C3—C4—C7—N2	−0.69 (18)
C2—C3—C4—C7	177.96 (13)		

### Hydrogen-bond geometry (Å, º)

**Table:**

*D*—H···*A*	*D*—H	H···*A*	*D*···*A*	*D*—H···*A*
O1—H1···Cl1^i^	0.82	2.34	3.147 (1)	167
N1—H1*N*···Cl1^ii^	0.91 (1)	2.14 (1)	3.040 (1)	173 (1)
C6—H6···N2^iii^	0.93	2.59	3.516 (2)	173
C7—H7···Cl1^iv^	0.93	2.81	3.697 (1)	160
C9—H9*B*···Cl1^v^	0.96	2.81	3.713 (2)	158

Symmetry codes: (i) −*x*+1, *y*+1/2, −*z*+3/2; (ii) *x*, *y*, *z*−1; (iii) −*x*+1, *y*−1/2, −*z*+1/2; (iv) −*x*+1, −*y*, −*z*+1; (v) −*x*, −*y*, −*z*+1.
